# Ovarian cancer G protein-coupled receptor 1 inhibits A549 cells migration through casein kinase 2α intronless gene and neutral endopeptidase

**DOI:** 10.1186/s12885-022-09257-1

**Published:** 2022-02-05

**Authors:** Adhikarimayum Lakhikumar Sharma, Puyam Milan Meitei, Takhellambam Chanu Machathoibi, Naorem Tarundas Singh, Thiyam Ramsing Singh, Lisam Shanjukumar Singh

**Affiliations:** 1 Cancer and Molecular Biology Division, Department of Biotechnology, Manipur University, Canchipur- 795003, Imphal, Manipur India; 2 Present Address: Center for Translational Medicine, Thomas Jefferson University, 1020 Locust Street, Philadelphia, PA 19107 USA

**Keywords:** GPR68, Ovarian cancer G protein-coupled receptor 1, Casein kinase 2α, Intronless, Neutral endopeptidase

## Abstract

**Background:**

We have previously reported that a new intronless gene for casein kinase 2α (CK2α), CSNK2A3, is expressed in human cells. The promoter of the well-known CK2α, CSNK2A1, displays characteristics of a housekeeping gene, whereas CSNK2A3 has a characteristic of a regulated promoter with two TATA boxes and a CAAT box. GPR68, a family of the G protein-coupled receptors, is also known as ovarian cancer G protein-coupled receptor 1 (OGR1). In the current study, we analyzed the roles of CK2α genes and neutral endopeptidase (NEP), a key enzyme that influences a variety of malignancies, in the OGR1-induced inhibition of A549 cell migration.

**Methods:**

We analyzed the transcript expressions of both the CK2α genes (CSNK2A1 and CSNK2A3) and NEP upon OGR1 overexpression. Protein expression of CK2α and NEP were also analyzed. We further elucidated the functional roles of both CK2α and NEP in the OGR1-induced inhibition of A549 cell migration in vitro using a wound-healing assay. We also analyzed the molecular mechanisms involved in the OGR1-induced inhibition of lung cancer cell migration.

**Results:**

The findings of this study showed that OGR1 upregulated the expression of CSNK2A3 but not CSNK2A1 in the A549 cells. The findings further suggested OGR1 also upregulates the expression of NEP. The OGR1-induced inhibition of A549 cell migration was abrogated completely by inhibition of CK2α activity, whereas partial abrogation (~ 30%) was observed in the presence of NEP inhibition. The results also revealed that OGR1 regulates CSNK2A3 via activation of Rac1/cdc42 and MAPKs pathways. CK2 is ubiquitously expressed, and in contrast, is believed to be a constitutively active enzyme, and its regulation appears to be independent of known second messengers.

**Conclusion:**

In the current study, we report for the first time the OGR1-induced regulation of CSNK2A3, CK2αP, and NEP in A549 cancer cells. Our study also decoded the downstream cellular proteins of OGR1 as well as the molecular mechanism involved in OGR1-induced inhibition of A549 cell migration. The findings of this research suggest the potential therapeutic targets to inhibit lung cancer progression.

**Supplementary Information:**

The online version contains supplementary material available at 10.1186/s12885-022-09257-1.

## Introduction

GPR68 was first cloned from an ovarian cancer cell. Therefore, it is also known as ovarian cancer G protein-coupled receptor 1 (OGR1) [1]. OGR1 and related subfamily members mediate the functions of several lysophospholipids, which include endothelial barrier function, endothelial cell proliferation, migration, and tube formation, T cell migration, glucocorticoid-induced thymocyte apoptosis, and globoid cell formation [2–6]. The OGR1 is expressed at lower levels in metastatic compared with primary prostate cancer tissues [7]. Using an orthotopic mouse metastasis model, we have shown that OGR1 is a metastasis suppressor gene for prostate cancer [6]. Further, studies have also reported that OGR1 inhibits breast and ovarian cancer cells in vitro when it is reexpressed in cancer cells [8, 9]. On the other hand, our previous study revealed that OGR1 has an inhibitory effect on prostate cancer tumorigenesis when expressed in host/stromal cells using the OGR1-knockout TRAMP mice prostate cancer model [10]. Further, we have also demonstrated that OGR1 significantly inhibited melanoma tumorigenesis in OGR1 knockout mice [11]. Another study by Horman SR, et al. reported that murine colon tumor implants in OGR1 knockout mice displayed delayed tumor growth [12]. Therefore, these previous reports indicate that in contrast to OGR1’s tumor-suppressing role in tumor cells, host cell OGR1 may be involved in and/or required for tumor growth. Moreover, the signaling pathway of OGR1 in inhibition of metastasis is not clearly understood. One previous report has revealed that OGR1 induced activation of Rho but down-regulated Rac1 in breast cancer cell lines [9]. Therefore, it is important to investigate the downstream cellular proteins of OGR1 in its function as a metastasis suppressor gene.

Casein kinase 2α (CK2α) acts as a regulator of several hallmarks of cancer cell behavior [13–15]. CK2α gene may potentially be induced or repressed by several master regulators of developmental pathways [16–18]. We reported for the first time that an intronless gene of CK2α (CSNK2A3) is expressed in human megakaryocytic cells [18]. Recently other studies revealed that CSNK2A3 is expressed in 293 T, A549, and NIH-3T3 cells and further polymorphism of CSNK2A3 plays oncogenic roles in lung cancer [19]. CK2 proteins are upregulated in the human tumors tested so far, suggesting their important role in cancer progression [20, 21]. In cancer, CK2 is proposed to regulate essential cellular processes such as cell growth [22], cell proliferation [23], cell survival [24], cell morphology [25], cell transformation [26, 27] and angiogenesis [28]. Although multiple layers of regulation of CK2α expression have been observed [21, 29], reports on the regulation of CK2α expression are very limited. The original view in the literature is that CK2 is predominantly regulated post-transcriptionally; however, recent studies strongly suggest that regulation at the transcriptional level is also important in some cancers [30]. Recently, Das, N et al. reported that Estrogen receptor alpha (ERα) transcriptionally activates CKα [31]. Unpredictably, some cancers show under-expression of CK2 transcripts in breast, ovarian, and pancreatic cancer [30]. Furthermore, CK2 transcript levels could have a prognostic value in cancers (e.g., CK2α in squamous cell carcinoma of the lung). For the most part, high levels of CK2 transcript correlate with lower overall survival (e.g., breast and ovarian cancer, glioblastoma, kidney, and liver cancer)[30, 32–34]. However, in lung adenocarcinoma, high levels of CSNK2A2 (CK2α′) and CSNK2A3 correlate with higher survival rates [21, 30]. Similar to lung adenocarcinoma, overexpression of CSNK2A3 in renal clear cell carcinoma led to increased survival [21]. From the above data, it is not clear whether CK2 is an anti-cancer or pro-cancer molecule.

Neutral endopeptidase 24.11 (NEP, neprilysin, enkephalinase, CD 10) is a widely distributed membrane enzyme, occurring on a variety of cells [35]. The biological and regulatory effects of NEP are presumed only to result from its enzymatic function [36, 37]. However, recent data suggest that NEP protein expression in itself can affect signal transduction pathways that regulate cell growth [38, 39] and apoptosis [40]. Tokuhara et al. reported that tumors with high NEP and low CD13 were associated with a better prognosis [41]. Gurel et al. reported that in lung squamous cell carcinomas, both tumoral and stromal NEP expression were unfavorable prognostic factors, while in non-squamous cell carcinomas tumoral NEP expression was a favorable prognostic factor [42]. Therefore, the prognostic value of tumoral NEP for early-stage lung adenocarcinoma remains unknown. In the current study, we aim to identify the key cellular protein(s) involved in the inhibition of cell migration induced by a metastasis suppressor gene, OGR1.

## Material and methods

### Cell culture and reagents

The adenocarcinoma human alveolar basal epithelial (A549) cells were procured from the National Centre for Cell Science (NCCS) Pune, India. The cells were maintained at 37 °C, 5% CO2 in F-12 K Medium (Kaighn's Modification of Ham's F-12 Medium) supplement with 10% fetal bovine serum (FBS) and 1% penicillin/streptomycin. All the cell culture media and serum were procured from Gibco (Waltham, MA, USA) while the antibiotics (penicillin/streptomycin) were procured from Thermo Fisher Scientific (Waltham, MA, USA). The antibodies for ERK, phospho-ERK, JNK, phospho-JNK, p38, and phospho-p38 were purchased from Cell Signaling Technology (Danvers, MA, USA). Antibodies for CK2 (Santa Cruz Biotechnology, Dallas, TX, USA), OGR1 (Cat No. 72500, abcam, Cambridge, UK), NEP (Cat no. sc-9149, Santacruz Biotechnology, Dallas, TX, USA), and β-actin (Cat no. sc-47778, Santa Cruz Biotechnology, Dallas, TX, USA) were also procured. Specific inhibitors for JNK; SP600125, for p38; SB203580 inhibitors were procured from the Abcam (Cambridge, UK) while the specific inhibitor for ERK, FR180204, silmitasertib (CX-4945), the specific inhibitor for CK2α, DL-Thiorphan, the specific inhibitor for NEP and Pertussis toxin (PTX) were procured from Sigma-Aldrich (Burlington, MA, USA).

### Plasmid construction and gene transfer

pcDNA3.1-OGR1 was constructed as described previously [6]. Briefly, the OGR1 coding sequence fragment (≈ 1.2 kb) was amplified and then cloned into pcDNA3.1 (puromycin) by EcoRI and HindIII digestion. The plasmid construct was transformed in DH5α-competent E. coli, and selected colonies were then cultured. Plasmid DNA was purified using QuickLyse Miniprep Kit (Qiagen, Hilden, Germany). The construct containing OGR1 was further confirmed by sequencing using the ABI Prism 377 Automated DNA Sequencer (Applied Biosystems, Waltham, MA, USA), and the DNA sequences and reading frames were further verified. The plasmids pcDNA3.1-cdcT17N and pcDNA3.1-RacT17N were procured from Addgene (Watertown, MA, USA). A549 cells were transiently transfected with 1.0–1.5 µg plasmids; pcDNA3.1-OGR1 and empty vector (pcDNA3.1); or co-transfected empty vector or pcDNA3.1-OGR1 with pcDNA3.1-cdcT17N and pcDNA3.1-RacT17N using Lipofectamine 2000 (Thermo Fisher Scientific, Waltham, MA, USA) according to the manufacturer’s protocol. The final concentration of the SP600125, SB203580, and FR180204 was 20 µM, and for PTX, silmitasertib (CX-4945) and DL-Thiorphan were 100 nM, 2.5 µM, and 10 µM, respectively. All the inhibitors were added to the cells after 5 h of transfection. Treated cells were used for the following experiments 48 h after transfection.

### Construction, packaging, and infection of lentivirus vector

To knock down the expression of OGR1, the selected interfering [short hairpin (shRNA)] sequence 5´-CCGGCCACCGTTGTCACAGACAATGCTCGAGCATTGTCTGTGACAACGGTGGTTTTTG-3´ (adding AgeI and EcoRI sites at 5´- and 3´- ends respectively) targeting the untranslated region of OGR1 was cloned into pLKO.1 vector (Addgene Plasmid No. 10878) after the oligonucleotides were annealed. The lentiviral vectors pLKO.1-shOGR1 and the negative control (NC) lentivirus pLKO.1-shNC were transfected with the corresponding helper plasmids into 293 T cells using Lipofectamine® 2000 (Invitrogen, Waltham, MA, USA). The supernatant containing viral particles was collected after 48 h of transfection. The viral particles were used to transduce A549 cells for 48 and 72 h.

### RNA isolation, cDNA synthesis, and PCR amplification to measure the expression of casein kinase alpha and neutral endopeptidase

Total RNAs were extracted from 8 × 10^5^ cultured cells using an RNA isolation kit (Qiagen, Hilden, Germany) according to the manufacturer’s instruction. The isolated RNAs were analyzed for their integrity, purity, and yield. Using the isolated RNAs as the template, first-strand complementary DNA (cDNA) was synthesized using M-MuLV Reverse Transcriptase (NEB, Ipswich, MA, USA). Briefly, the extracted RNA (≈ 3 µg) was reverse transcribed in a total volume of 20 µl with 350 µM dNTP, 50 µM oligo(dT), 10X M-MuLV buffer, 200U RNase inhibitors, and 200U M-MuLV reverse transcriptase. All the reagents were mixed and incubated at 42ºC for 1 h followed by 65ºC for 20 min. PCR was then performed using Platinum Taq DNA polymerase (Invitrogen, Waltham, MA, USA). The reaction volume was 50 µl, containing 10X PCR buffer, 350 µM dNTP mixtures, 0.4 mM of each primer, and 5U Taq DNA polymerase. Cycling conditions were 94ºC for 4 min and then 25 cycles of (94ºC 30 s, 55ºC 30 s, and 72ºC 45 s) followed by 72ºC for 10 min. Quantitative PCR (qPCR) was performed with primers obtained from Xcerlis genomics Laboratory (Ahmedabad, India). The primer sequences were for OGR1-specific primers; forward primers (5′-CTGTCCTGCCAGGTGTGCGG-3′) and reverse primers (5′-CACGCGGTGCTGGTTCTCGT-3′). The β2-microglobulin housekeeping gene (NM_004048) was used as a loading control. The primer sequences used to amplify the β-microglobulin were (forward) 5′- GAGCCTCGCCTTTGCCGATG-3′ and (reverse) 5′-CGATGCCGTGCTCGATGGGG-3′. The primer sequences for CSNK2A1 were (forward) 5′-CCAAACATCAAGTCCAGCTTTGTC-3′ and (reverse) 5′-ACCTCGGCCTAATTTTCGAACCA-3′ and for CSNK2A3 were (forward) 5′-ATTGCTCCCCACTCCATCGC-3′ and (reverse) 5′-ACCTCGGCCTAATTTTCGAACCA-3′. The primer sequences for NEP were (forward) 5′-GCCTCTCGGTCCTTGTCCTGC-3′and (reverse) 5′-ACGGGAGCTGGTCTCGGGAA-3′.

### Cell lysate preparation and western blotting

Total cell lysates were prepared with radioimmunoprecipitation assay (RIPA) buffer. Briefly, cells were lysed in 300 µl of RIPA buffer (10 mM Tris–HCl [pH 7.4], 150 mM NaCl, 1% Triton X-100, 5 mM EDTA, 1% sodium deoxycholate, 0.1% SDS, 1.2% aprotinin, 5 μM leupeptin, 4 μM antipain, 1 mM phenylmethylsulfonyl fluoride [PMSF], and 0.1 mM Na3VO4, sodium orthovanadate). The cell lysate was then centrifuged at 17,000xg for 1 h and protein concentration in the samples was normalized using standard BCA assay (Thermo Fisher Scientific, Waltham, MA, USA). After normalization, equal amounts of protein samples were mixed with 4X Laemnli sample buffer, resolved by SDS-PAGE on 10% gels, and then transferred onto PVDF membranes. The membranes were blocked with 5% non-fat milk for 1 h, incubated with primary antibodies (1:2000 dilution) at 4 °C overnight, and then with secondary antibody (1:6000 dilution) for 1 h at room temperature. The blots were detected using a chemiluminescent ECL system (GE Healthcare, Chicago, IL, USA). Blot images were captured using ChemDoc (BioRad, Hercules, CA, USA).

### Wound-healing assay

To analyze the effect of OGR1 and CK2α on cell migration, A549 cell was seeded to obtain 70–90% confluency at the time of transfection. Cells were transfected with an empty vector (pcDNA3.1) or pcDNA3.1-OGR1. Inhibitors silmitasertib (CX-4945) and DL-Thiorphan were treated after 5 h of transfection. After treatment with inhibitors, cell-free areas were created (scratched) in a confluent monolayer cell using 10 µl filter tips. Cell migration was observed under the microscope, and the image was taken at 0, 12, 24, and 48 h).

### Statistical analysis

Data were analyzed using Microsoft Excel or GraphPad Prism 8.0 (GraphPad Software, San Diego, CA, USA). Experimental data are presented as the mean ± SD of at least three independent experiments. *p* < 0.05 was considered significant; *p* values were defined as ∗ *p* < 0.05 and ∗  ∗ *p* < 0.01.

## Results

### OGR1 regulates the expression of CSNK2A3 and NEP but not CSNK2A1

**Fig. 1:**
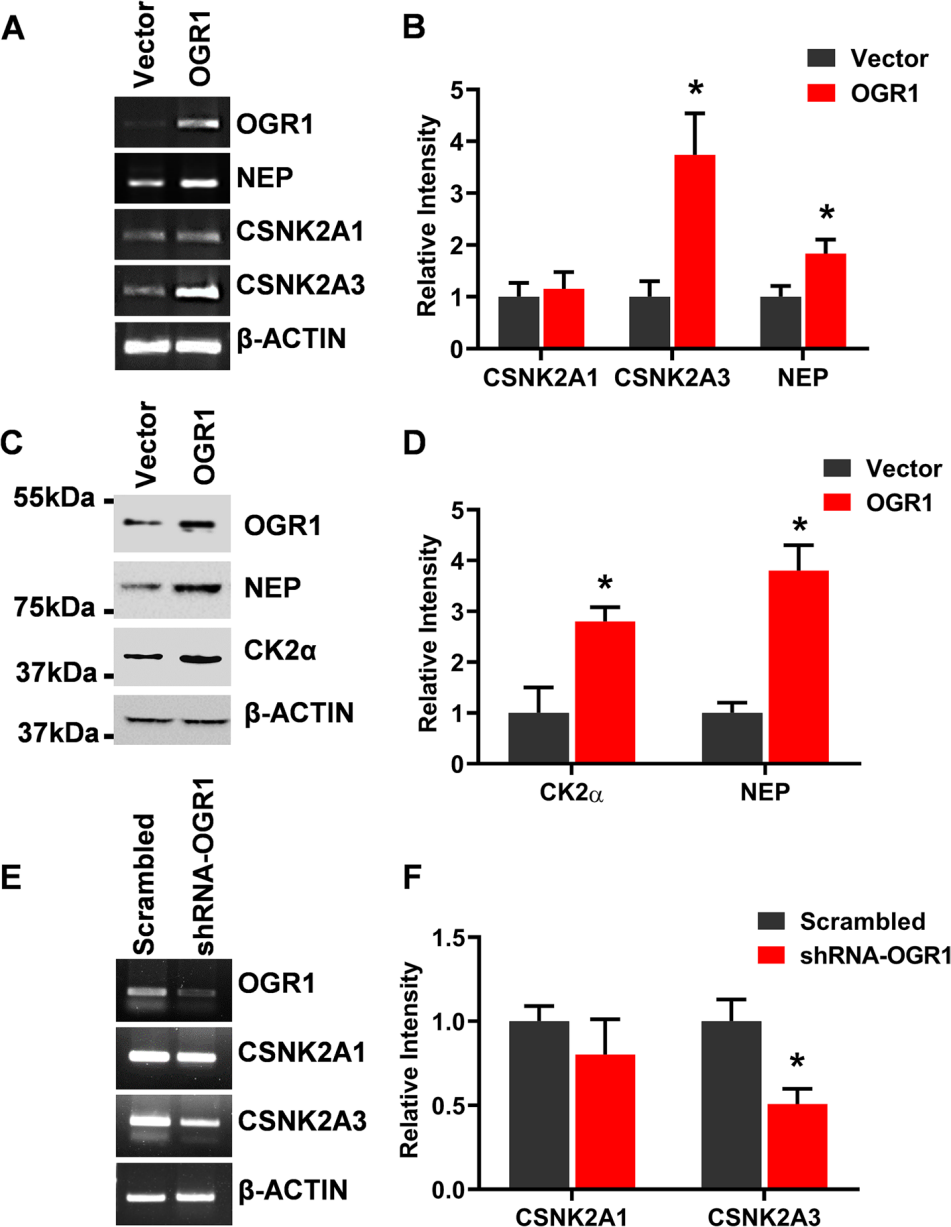
OGR1 upregulates CSNK2A3 (CK2α intronless gene) and NEP but not CSNK2A1 in A549: **A** A549 cells were transfected with pcDNA3.1-OGR1 (OGR1) or pcDNA3.1 (Vector), transcript expressions of CSNK2A1, CSNK2A3, and NEP were analyzed in the semi-quantitative RT-PCR. β-actin was used as a control for equal loading. **B** DNA bands intensities for CSNK2A1, CSNK2A3, NEP were analyzed by using ImageJ software and represented graphically after normalization to actin. **C** Protein expressions of NEP and CK2α were assayed by Western blotting with specific antibodies and β-actin was used as a control for equal loading, **D** protein band intensities were analyzed by using ImageJ software, and represented graphically after normalization to actin. **E** Transcript expression of CSNK2A1 and CSNK2A3 were analyzed after OGR1 knockdown in A549 cells by expressing shRNAs against OGR1, cells expressing scrambled shRNA as control. **F** DNA band intensities for CSNK2A1 and CSNK2A3 were analyzed by using ImageJ software and represented graphically. The display of cropped gels and blots are to improve the clarity and conciseness of the presentation. However, full length gels and blots are also included in supplementary file 2. Replicate of NEP and actin blots are also included in supplementary file 2. Error bars represent the Mean ± SD of three independent experiments. The *p*-value of statistical significance was set as *p* < 0.05 (*)

To investigate the downstream molecules involved in the OGR1-induced inhibition of cancer cell migration, OGR1 was over-expressed transiently in A549 cells for 48 h, and transcript expression of CSNK2A1, CSNK2A3, and NEP were analyzed using semi-qPCR. Initially, OGR1 over-expression in A549 was confirmed by semi-qPCR. The results showed that OGR1 strongly upregulates transcript expression of CSNK2A3 (CK2α intronless gene, CK2αP) and NEP. However, OGR1 does not affect the expression of CSNK2A1 in A549 cells significantly (Fig. 1A and B). Further, protein expression of CK2α and NEP were analyzed by immunoblotting upon OGR1 over-expression in A549 cells using respective specific antibodies (Fig. 1C and D). The over-expression of OGR1 protein was confirmed using a specific antibody against OGR1. The specific antibody for CK2α recognizes proteins of both CSNK2A1 and CSNK2A3 since there are only four amino acids different in the sequence of the two genes [18]. Taken together, the results of transcript expression of CK2α genes (CSNK2A1 and CSNK2A3) and protein expression of CK2α indicated that the increase in the protein of CK2α in immunoblotting may be the product of CSNK2A3 (CK2αP). The immunoblotting against NEP confirmed that OGR1 over-expression cells increased NEP expression in A549 cells (Fig. 1C**)**. Further, to validate the finding, OGR1 was knocked down by shRNA targeting OGR1. As an experimental control, cells were infected with a lentiviral vector expressing scrambled shRNA. Scrambled shRNA was checked and confirmed for its neutrality towards OGR1 and other cellular genomes. Based on semi-qPCR, a significant OGR1 knockdown was evident when compared with scrambled control (Fig. 1E). The depletion of OGR1 resulted in a significant decrease in CSNKA3 expression (Fig. 1E and F). Thus, the overall results indicated that OGR1 upregulates the expression of CSNK2A3 and NEP, but not CSNK2A1 in lung cancer cells. Our finding is the first report to demonstrate that the expression of CSNK2A3 is regulated. There is a very limited report of how CK2α gene expression is regulated, despite many reports on aberrant expression in cancer.

### CK2αP is upstream of NEP in the OGR1 signaling pathway

**Fig. 2:**
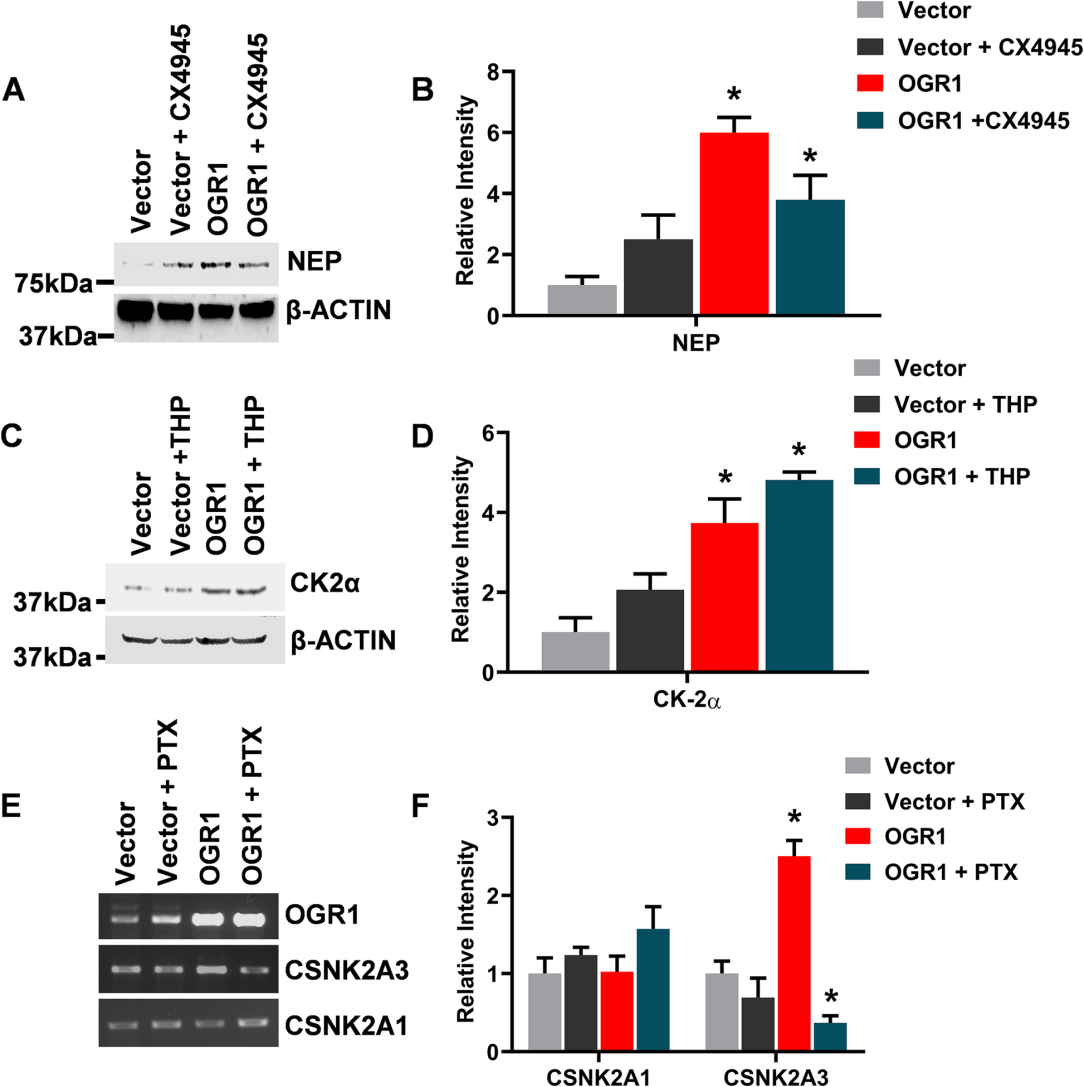
CK2 is upstream of NEP in the OGR1 signaling pathway: Western blot assay of NEP and CK2αP expression in presence or absence of specific inhibitors. **A** A549 cells were transfected with pDNA3.1-OGR1 (OGR1) or pcDNA3.1 (Vector) and NEP expression was analyzed with anti-NEP in the presence or absence of CX-4945, CK2 inhibitor (CX4945), and **B** protein band intensities were analyzed by using ImageJ software, and represented graphically after normalization to actin. **C** Expression of CK2α was analyzed with anti CK2α in the presence or absence of NEP inhibitor, thiorphan (THP), and **D** protein band intensities were analyzed by using ImageJ software and represented graphically after normalization to actin. **E** Semi-qPCR showing the expression of NEP and CK2 in the presence or absence of Gαi inhibitor PTX and **F** intensities of DNA bands were analyzed by using ImageJ software and represented graphically. The display of cropped gels and blots are to improve the clarity and conciseness of the presentation. However, full length gels and blots are also included in supplementary 2. Replicates of NEP and actin blots are also included in supplementary file 2. Error bars represent the Mean ± SD of three independent experiments. The *p*-value of statistical significance was set as *p* < 0.05 (*)

To investigate whether CK2αP is upstream of NEP or vice-versa in the OGR1 signaling pathway, we assessed the expression of both the proteins in the presence of either the specific chemical inhibitor of CK2; CX-4945; and immunoblotting against NEP or specific chemical inhibitor of NEP, thiorphan, and immunoblotting against CK2α, after A549 cells were transiently transfected with OGR1 or empty vector (control). Interestingly, the results showed that in the presence of CX-4945, increased expression of NEP induced by OGR1 is abrogated to a similar level of control cells (Fig. 2A and B). We have also performed a knockdown experiment to validate our inhibitor result (Fig. S1). However, thiorphan did not affect the expression of CK2α protein in A549 cells (Fig. 2C and D). These results indicated that CK2αP is upstream of NEP in the signaling pathway of OGR1. It was previously reported that CK2α regulates NEP activity by phosphorylating at the cytoplasmic tail [43].

Further, previously we have reported that OGR1 inhibits PC3 cell migration via Gαi activation [6]. Therefore, to investigate whether OGR1 induced up-regulation of CSNK2A3 in A549 is dependent on the activation of Gαi, we analyzed the transcript expression of CSNK2A3 in the presence or absence of pertussis toxin (PTX) upon OGR1 over-expression or empty vector. We used the transcript expression of CSNK2A1 as a control. The result revealed that the presence of PTX abrogated the OGR1-induced up-regulation of expression of CSNK2A3 (Fig. 2E and F**)**.

### CK2αP is functionally involved in OGR1-induced inhibition of lung cancer migration

**Fig. 3:**
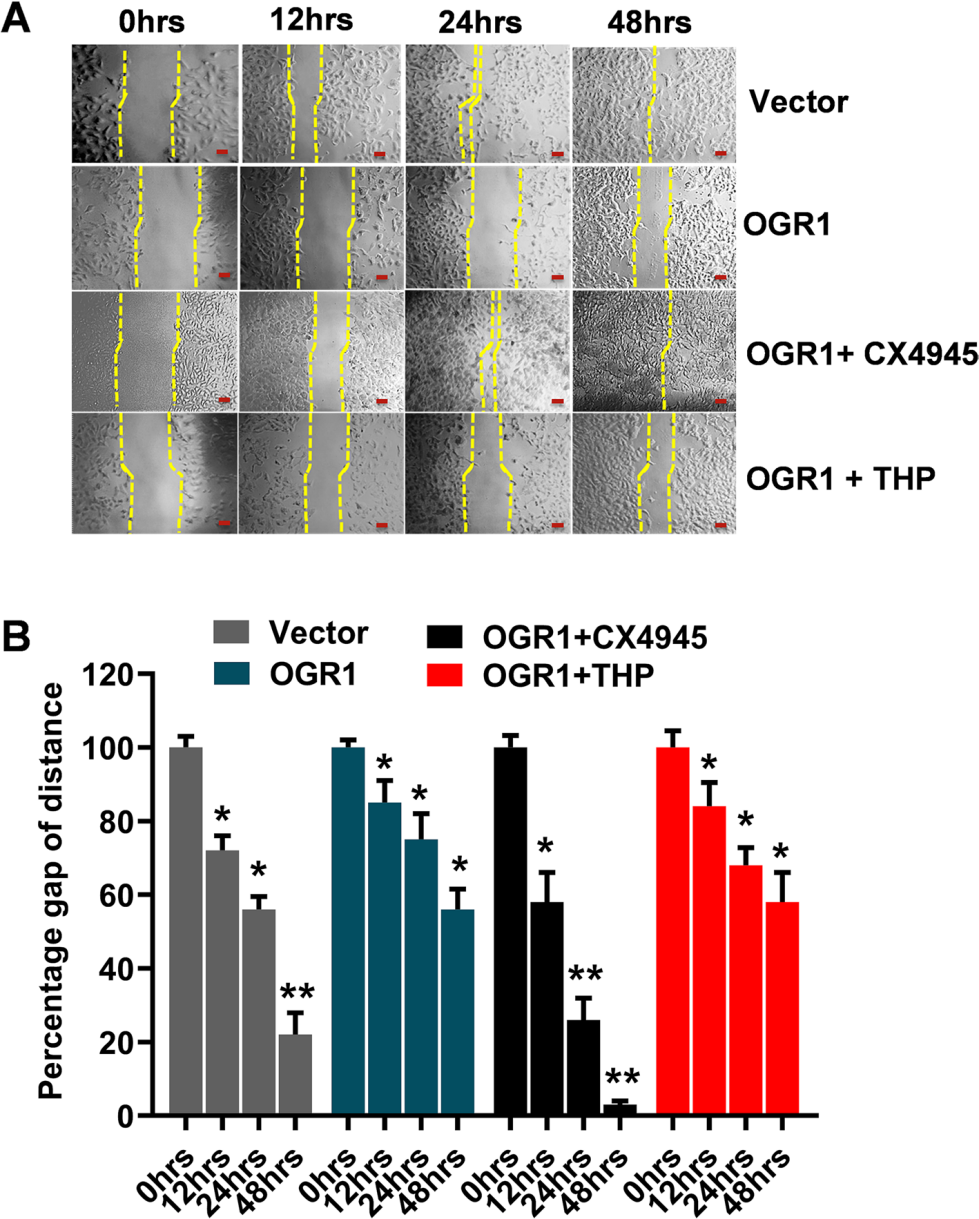
OGR1 inhibits A549 cell migration via CK2α: **A** OGR1 inhibits A549 cell migration, but the presence of CK2 inhibitor, CX4945, abrogated the effect of OGR1 but not the presence of NEP inhibitor, thiorphan (THP). A549 cells were transfected with pDNA3.1-OGR1 or pcDNA3.1 (Vector) in the presence of CK2 and NEP inhibitors. Cell migration was analyzed, and the image was taken hourly. **B** To measure the area of the wound, the length of scratches was measured on the light microscope and plotted on the graph. The wound area of control 0 h was taken as 100%, and the red line in Fig. 3A indicates the scale of 100 µm. Error bars represent the Mean ± SD of three independent experiments. The *p*-value of statistical significance was set as *p* < 0.05 (*) or 0.01 (**)

We have previously demonstrated that OGR1 is a metastatic suppressor gene in vitro as well as in vivo using an orthotopic mouse metastasis model [6]. In the current study, we found that OGR1 induced increased expression of CK2αP and NEP in (at both) transcript and protein levels. Therefore, in the current study, we investigated the functional roles of both CK2α and NEP in the OGR1-induced inhibition of A549 cell migration in vitro using a wound-healing model in the presence or absence of an inhibitor of CK2α and NEP. The results showed that OGR1 inhibits the migration of A549 cells. In the presence of the CK2-specific inhibitor, CX-4945, the OGR1-induced inhibition of A549 cell migration was abrogated completely (Fig. 3A and B**)**. We further confirm this with CK2 knockdown (Fig. S1). However, thiorphan decreased the OGR1-induced inhibition of A549 cell migration only by ~ 30%, indicating there may be molecule (s) other than NEP through which CK2αP induces inhibition of cancer cell migration following the action of OGR1.

### Involvement of Rac1 and cdc42

**Fig. 4:**
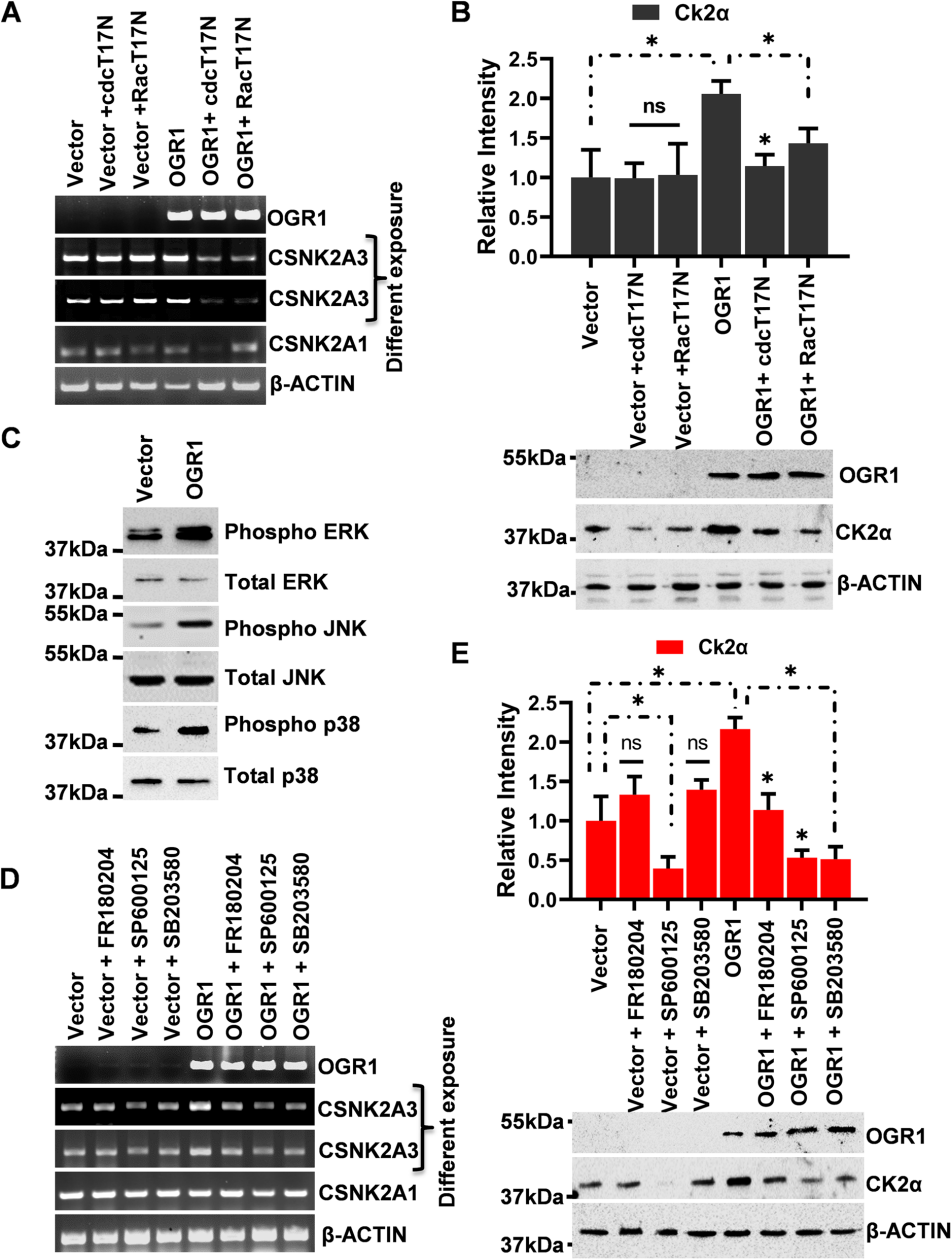
Role of Rac/CDC42 and MAPK pathways in the regulation of CK2α genes induced by OGR1: Inhibition of Rac activity abrogated up-regulation of CSNK2A3 induced by OGR1. A549 cells were co-transfected with OGR1 or pcDNA3.1 (Vector) and negative dominant mutants of Rac (pcDNA3.1-RacT17N) or negative dominant mutants of CDC42 (pcDNA3.1-cdcT17N). **A** Semi-quantitative RT-PCR was performed to analyze CSNK2A1 and CSNK2A3 transcript expression. β-actin was used as a control for equal loading. **B** Western blot assay was further analyzed to check the protein expression of CK2α. MAPK pathways in the regulation of CK2α genes induced by OGR1. A549 cells were also transfected with pDNA3.1-OGR1 or pcDNA3.1 (Vector) and activation of MAPK families, **C** ERK, JNK, p38, were analyzed by western blotting. **D and E** Semi-quantitative RT-PCR and western blot were performed to analyze CSNK2A3 transcript and CK2αP expression, respectively, in the presence of specific MAPK inhibitors. β-actin was used as a control for equal loading. The display of cropped gels and blots are to improve the clarity and conciseness of the presentation. However, full length gels and blots are also included in supplementary file 2. Error bars represent the Mean ± SD of three independent experiments. The *p*-value of statistical significance was set as *p* < 0.05 (*)

The known roles of the small GTPase, Rac1 and cdc42, as major drivers of cell motility prompted us to assess whether Rac1 and cdc42 are involved in the OGR1-induced up-regulation of CK2αP expression, which leads to inhibition of A549 cell migration. A549 cells were transiently transfected with OGR1 alone or co-transfected with cdcT17N (dominant-negative of cdc42) or RacT17N (dominant-negative of Rac1). Similarly, control A549 cells were also transiently transfected with an empty vector alone and co-transfected with cdcT17N or RacT17N. Initially, the over-expression of OGR1 in the OGR1-transfected cells was confirmed by semi-qPCR. The results showed that in the presence of cdcT17N and RacT17N, the OGR1-induced up-regulation of CSNK2A3 in A549 cells was significantly abrogated, suggesting that both small G proteins, cdc42 and Rac1, are involved in the OGR1-induced up-regulation of  CSNK2A3 in A549 cells (Fig. 4A**)**. These findings were further validated by western blotting (Fig. 4B).

### Involvement of MAPK pathway

In our quest to define the molecular mechanisms involved in the OGR1-induced inhibition of lung cancer cell migration, we evaluated the mitogen-activated protein kinase (MAPK) signaling pathway. Since the MAPK cascades are well documented for their roles in the transduction of extracellular signals to cellular responses such as cell proliferation, death, differentiation, migration, and invasion, we hypothesize that MAPK activation plays a key role in OGR1-mediated up-regulation of CK2αP expression to inhibit A549 cell migration. Initially, to test this hypothesis, OGR1 was over-expressed transiently in A549 cells and activations of three MAPK families; namely classical MAPK (extracellular signal-regulated kinase also known as ERK), C-Jun N-terminal kinase/ stress-activated protein kinase (JNK/SAPK), and p38 kinase were analyzed by western blotting. The results showed that OGR1 strongly increased the protein  level of phosphorylated ERK, JNK, and p38, whereas the protein level of total ERK, JNK, and p38 remained unchanged (Fig. 4C**).** Further to confirm the functional involvement of MAPK pathway in the OGR1-induced up-regulation of CK2αP, selective chemical inhibitors of ERK, Jun kinase (JNK), and p38 were treated in the OGR1 or empty vector-transfected A549 cells, and transcript and protein expression of CK2αP were analyzed. The results showed that the presence of selective inhibitors of MAPK, ERK, or JNK or p38 abrogated the OGR1-induced up-regulation of CSNK2A3 transcript and CK2αP expression (Fig. 4E and F**)**. Thus, the overall results indicated that OGR1 inhibits A549 cell migration via up-regulation of CK2αP expression through activation of MAPK pathways.

## Discussion

Unraveling the molecular mechanisms that control metastasis is crucial for cancer cure. Although OGR1 has been reported as a metastasis suppressor gene, the molecular mechanism is yet to be understood. The studies presented herein provide strong and compelling evidence for the important role of OGR1 in lung cancer. In this study, we demonstrated that OGR1 regulates the expression of a cellular enzyme which acts as a regulator of several hallmarks of cancer cell behavior, CK2α [13–15], and an important membrane enzyme, neutral endopeptidase 24.11 (NEP, neprilysin, enkephalinase, CD 10). To the best of our knowledge, our finding is the first report to demonstrate that the expression of CSNK2A3 (CK2αP) is regulated by a GPCR, OGR1. Our findings suggest that the aberrantly expressed transcript and/or protein of CK2α found in various cancer cells may be due to regulated CSNK2A3 (CK2αP) expression, which is potentially inducible or repressible by several master regulators of developmental pathways. The CSNK2A3 might have an advantage over the CSNK2A1 in cancer cells in which sophisticated lineage-specific gene expression is necessary [18]. However, further investigation is required to find out the expression of CSNK2A1 and CSNK2A3 in various cancer cells employing strategies that can distinguish expression from one another. It is important to mention that the mRNA sequence of CSNK2A3 is 99.7% homologous to CSNK2A3 [18].

Our results further showed OGR1 also upregulates expression of the transcript as well as the protein of NEP, a cell surface peptidase that is normally expressed by numerous tissues, including the prostate, kidney, intestine, endometrium, adrenal glands, and lung. Loss or decreases in NEP expression have been reported in a variety of malignancies [44]. Reduced NEP may promote peptide-mediated proliferation by allowing the accumulation of higher peptide concentrations at the cell surface and facilitating the development or progression of neoplasia. It has been shown that the effects of NEP are mediated by its ability to inactivate substrates such as bombesin and endothelin-1, but also through a direct protein–protein interaction with other proteins such as Lyn kinase (which associates with the p85 subunit of phosphatidylinositol 3-kinase [PI3-K] resulting in NEP-Lyn-PI3-K protein complex), ezrin/radixin/moesin (ERM) proteins, and the PTEN tumor suppressor protein. In addition, our study showed that CK2αP is upstream of NEP in the signaling pathway of OGR1, indicating CK2αP induces NEP gene expression as a result of OGR1’s action. The finding of CK2α’s regulation of NEP expression is of particular interest because of the role of CK2α in integrating essential cellular processes such as cell growth, cell proliferation, cell survival, cell morphology, cell transformation, and angiogenesis to proliferation and differentiation signals. In a variety of different cell types, peptide hormones, including epidermal growth factor, insulin, and the NEP substrate bombesin, increase CK2α activity. NEP plays a pivotal role in various cancers [37, 38]. It is previously reported CK2α inhibits NEP activity by phosphorylating the cytosolic domain [43]. Therefore, our results revealed another layer of fine-tuning of NEP activity by regulating its expression induced by CK2αP, the key cellular enzyme. Our results revealed that OGR1 upregulates the expression of CSNK2A3 transcript via Gαi activation.

Our finding also strongly suggested OGR1 inhibits cell migration via the expression of CK2αP (Fig. 3 and Fig.S1) Our finding supports the previous report that high levels of CK2αP correlate with higher survival rates of lung adenocarcinoma and renal clear cell carcinoma [29, 30]. The result also revealed that an inhibitor of NEP abrogates the OGR1’s inhibition of A549 cells to some extent (30%). Our results also revealed that CK2αP is upstream of NEP and inhibition of CK2 abrogated completely (100%) the OGR1-induced inhibition of A549 cell migration, whereas inhibition of NEP abrogated only 30%. Therefore, taken together, our results suggest there may be another cellular molecule other than NEP, which CK2αP targets/activates following OGR1 activation.

The result revealed that inhibition of Rac1 and cdc42 activities by respective negative dominant mutant proteins completely abrogated OGR1-induced up-regulation of CSNK2A3, whereas expression of CSNK2A1 is not affected (Fig. 4A and B). Cells spread by putting out extensions that contact the surface, form adhesions, and then exert tension to induce outward movement, a process reminiscent of the extensions and adhesions induced by the small GTP-binding proteins Rac and Cdc42 [45].

**Fig. 5:**
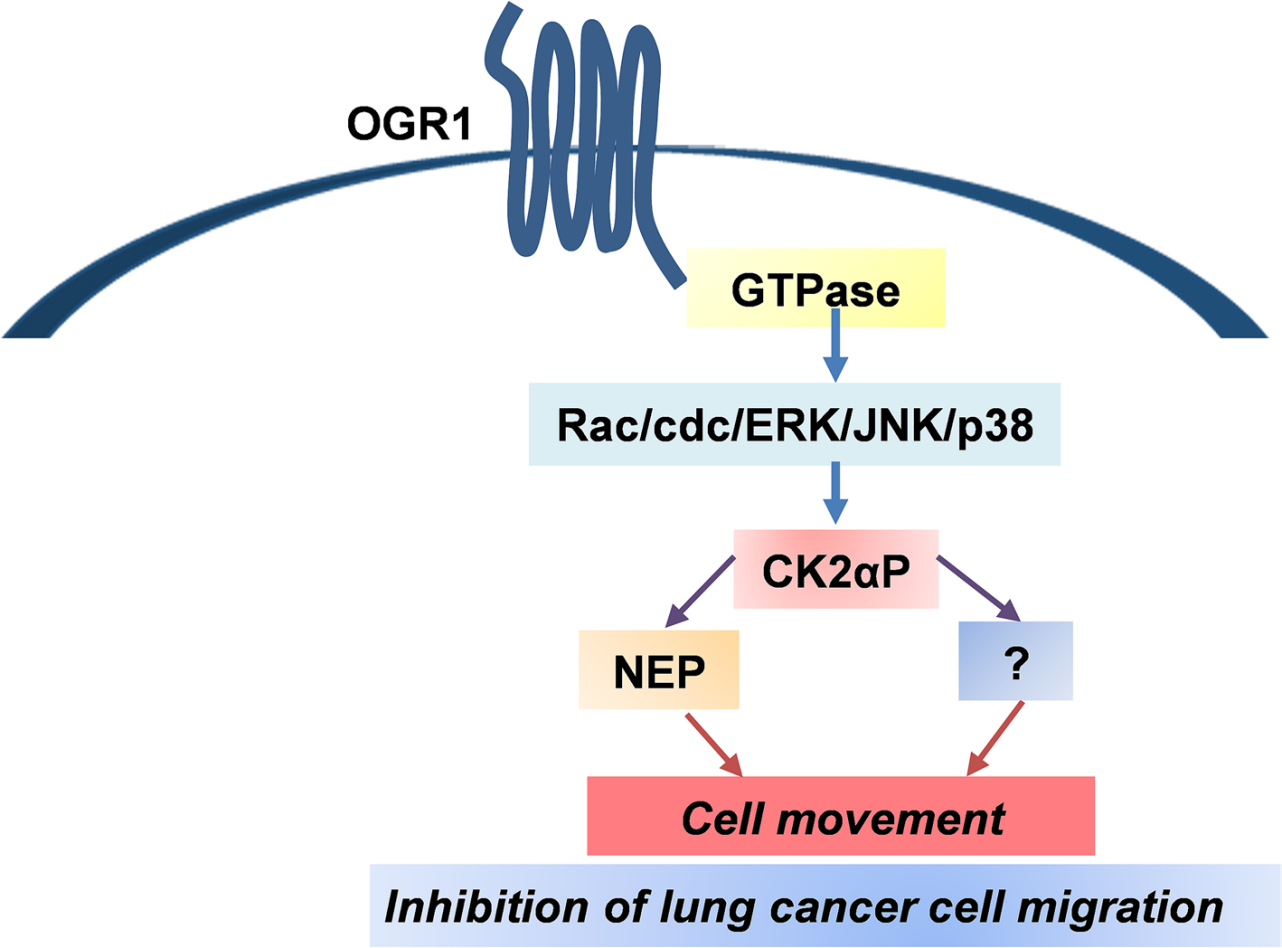
Speculative signaling pathways of OGR1 for inhibition of A549 cell migration are illustrated. OGR1 activates protein Gαi which leads to further activation of small G proteins, Rac1 and cdc42. Activations of Rac1 and cdc42 lead to activation of MAPK pathways which further increase expression of CK2αP and one or more other proteins), and finally, inhibit cell migration

Further, we assessed the role of MAPKs, extracellular signal-regulated kinase ERK, Jun kinase (JNK), and p38 since they have been shown to play a key role in the transduction of extracellular signals to cellular responses. The results clearly showed that inhibition of ERK, JNK, and p38 with the specific inhibitors completely abrogated OGR1-induced increased expression of CSNK2A3 transcript and CK2αP (Fig. 4D and E). Our findings unraveled the molecular mechanisms utilized by OGR1 to suppress metastasis. We propose a model to depict these multiple activities of OGR1 (Fig. 5). Overall results indicated that OGR1 regulates CK2αP expression via activation of small GTPase; Rac1 and cdc42; and MAPKs pathways.

## Conclusion

The overall findings of this study suggested that OGR1 regulates the expression of CK2αP and NEP in A549 cells. OGR1-induced up-regulation of CK2αP through activation of small GTPase, Rac1/cdc42, and ERK/JNK/p38 pathways are responsible for inhibition of cancer cell migration. However, OGR1 does not affect the expression of CSNK2A1. There is no previous report of how the expression of CK2α in cancer cells is regulated, although many studies have reported aberrant expression of the kinase in cancer. Therefore, our findings suggest that the aberrantly expressed transcript and/or protein of CK2α found in various cancer cells may be due to regulated CSNK2A3/CK2αP expression, which is potentially inducible or repressible by several master regulators of developmental pathways. CSNK2A3 might have an advantage over the CSNK2A1 in cancer cells in which sophisticated lineage-specific gene (s) expression is necessary.

## Supplementary Information

**Additional file 1.** (A) CK2 is upstream of NEP in the OGR1 signalling pathway. A549 cells were transfected with pcDNA3.1 vector (Empty vector) as control and co-transfected pcDNA3.1 (Empty vector) + psilencer-CSNK2A3 as positive control, pcDNA3.1-OGR1 and co-transfected pcDNA3.1-OGR1 + P silencer CSNK2A3. After 48hrs, total cell lysate was analysed by immunoblotting with NEP antibody. (B) OGR1 inhibits A549 cell migration via CK2α: OGR1 inhibits A549 cell migration, but knockdown of CSNK2A3 abrogated the effect of OGR1.

**Additional file 2.** All the original gel images and western blot figures.

## Data Availability

All data generated or analyzed during this study are included in this article.
