# Supplementary material for: Ovarian cancer G protein-coupled receptor 1 inhibits A549 cells migration through casein kinase 2α intronless gene and neutral endopeptidase
Source: BMC Cancer. 2022 Feb 5;22:143. doi: 10.1186/s12885-022-09257-1 (PMC8817493; doi:10.1186/s12885-022-09257-1)
Supplement: Supplementary file 1 — Additional file 1. (A) CK2 is upstream of NEP in the OGR1 signalling pathway. A549 cells were transfected with pcDNA3.1 vector (Empty vector) as control and co-transfected pcDNA3.1 (Empty vector) + psilencer-CSNK2A3 as positive control, pcDNA3.1-OGR1 and co-transfected pcDNA3.1-OGR1 + P silencer CSNK2A3. After 48hrs, total cell lysate was analysed by immunoblotting with NEP antibody. (B) OGR1 inhibits A549 cell migration via CK2α: OGR1 inhibits A549 cell migration, but knockdown of CSNK2A3 abrogated the effect of OGR1. [file 12885_2022_9257_MOESM1_ESM.pdf]

Supplementary file 1

Ovarian cancer G protein-coupled receptor 1 inhibits A549 cells Migration through Casein kinase 2α intronless gene and Neutral endopeptidase

Adhikarimayum Lakhikumar Sharma [1, 2], Puyam Milan Meitei [1], Takhellambam Chanu Machathoibi [1], Naorem Tarundas Singh [1], Thiyam Ramsing Singh [1], Lisam Shanjukumar Singh [1 *]

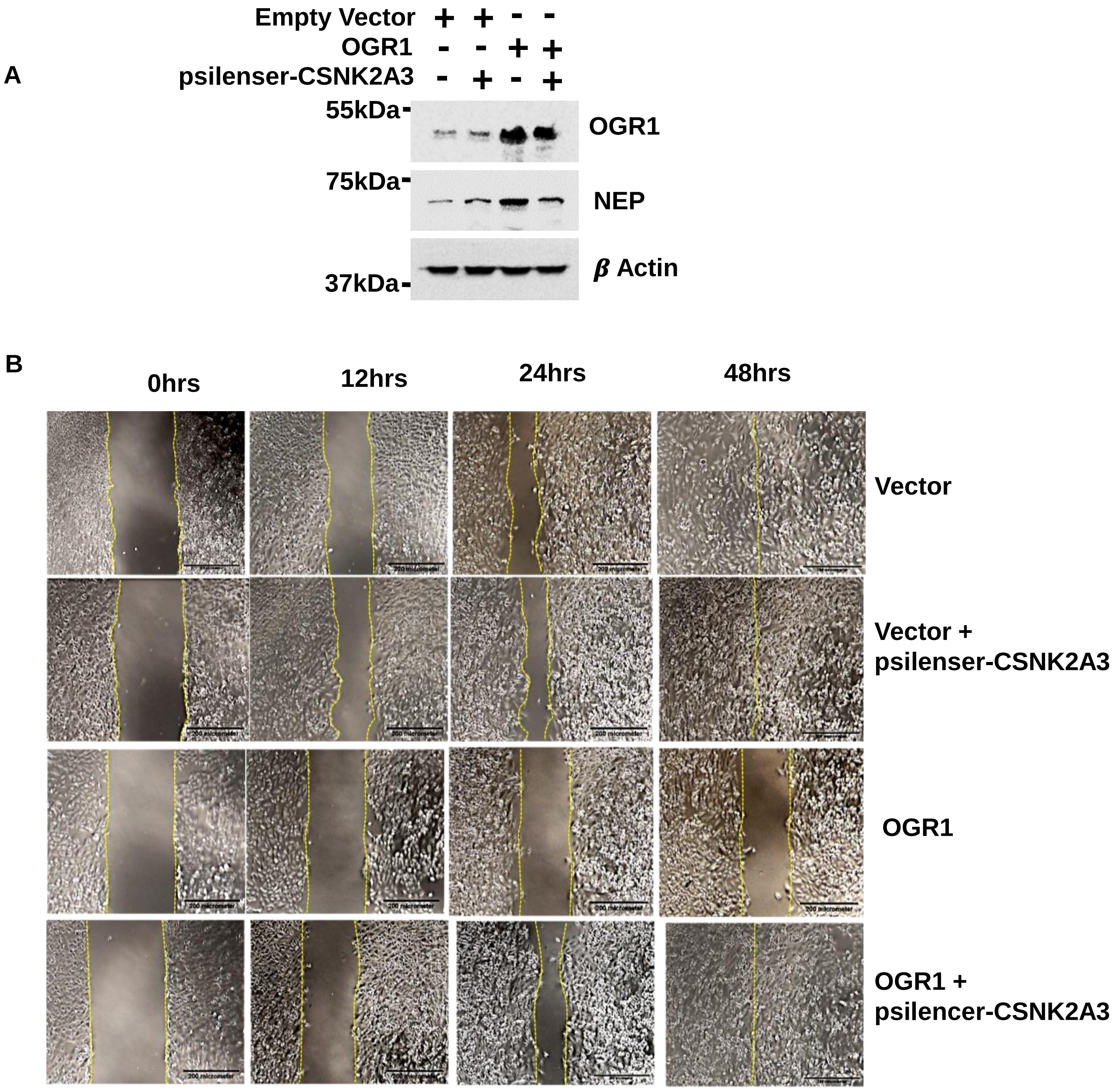

Supplementary Figure 1: (A) CK2 is upstream of NEP in the OGR1 signalling pathway. A549 cells were transfected with pcDNA3.1 vector (Empty vector) as control and co-transfected pcDNA3.1 (Empty vector) + psilencer-CSNK2A3 as positive control, pcDNA3.1-OGR1 and co-transfected pcDNA3.1-OGR1 + P silencer CSNK2A3. After 48hrs, total cell lysate was analysed by immunoblotting with NEP antibody. (**B) OGR1 inhibits A549 cell migration via CK2α:** OGR1 inhibits A549 cell migration, but knockdown of CSNK2A3 abrogated the effect of OGR1
