# Supplementary material for: Ovarian cancer G protein-coupled receptor 1 inhibits A549 cells migration through casein kinase 2α intronless gene and neutral endopeptidase
Source: BMC Cancer. 2022 Feb 5;22:143. doi: 10.1186/s12885-022-09257-1 (PMC8817493; doi:10.1186/s12885-022-09257-1)

Supplementary File 2

Ovarian cancer G protein-coupled receptor 1 inhibits A549 cells Migration through Casein kinase 2α intronless gene and Neutral endopeptidase

Adhikarimayum Lakhikumar Sharma [1, 2], Puyam Milan Meitei [1], Takhellambam Chanu Machathoibi [1], Naorem Tarundas Singh [1], Thiyam Ramsing Singh [1], Lisam Shanjukumar Singh [1 *]

Figure 1 Original blot and images

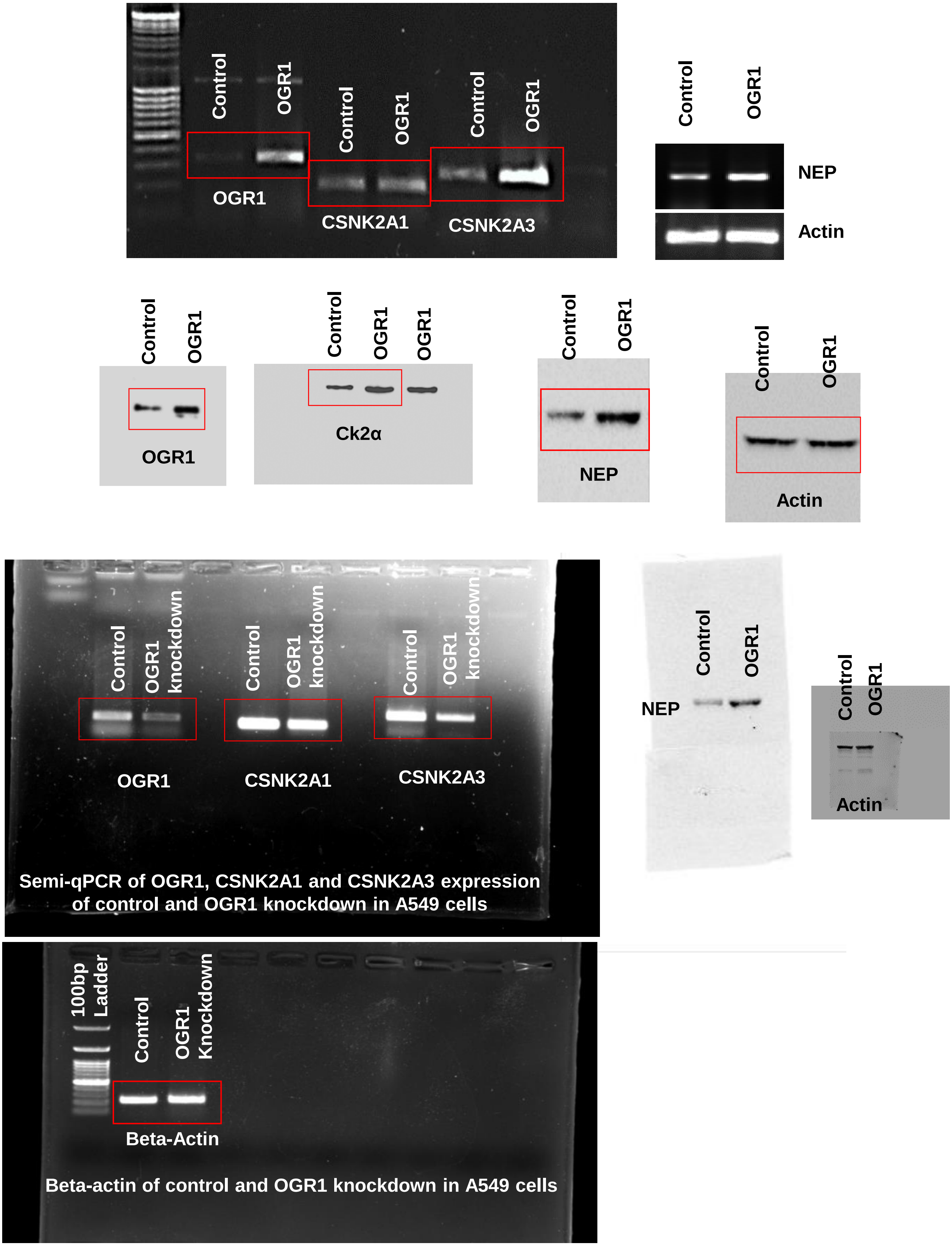

Ovarian cancer G protein-coupled receptor 1 inhibits A549 cells Migration through Casein kinase 2α intronless gene and Neutral endopeptidase

Adhikarimayum Lakhikumar Sharma [1, 2], Puyam Milan Meitei [1], Takhellambam Chanu Machathoibi [1], Naorem Tarundas Singh [1], Thiyam Ramsing Singh [1], Lisam Shanjukumar Singh [1 *]

Figure 2 Original blot and images

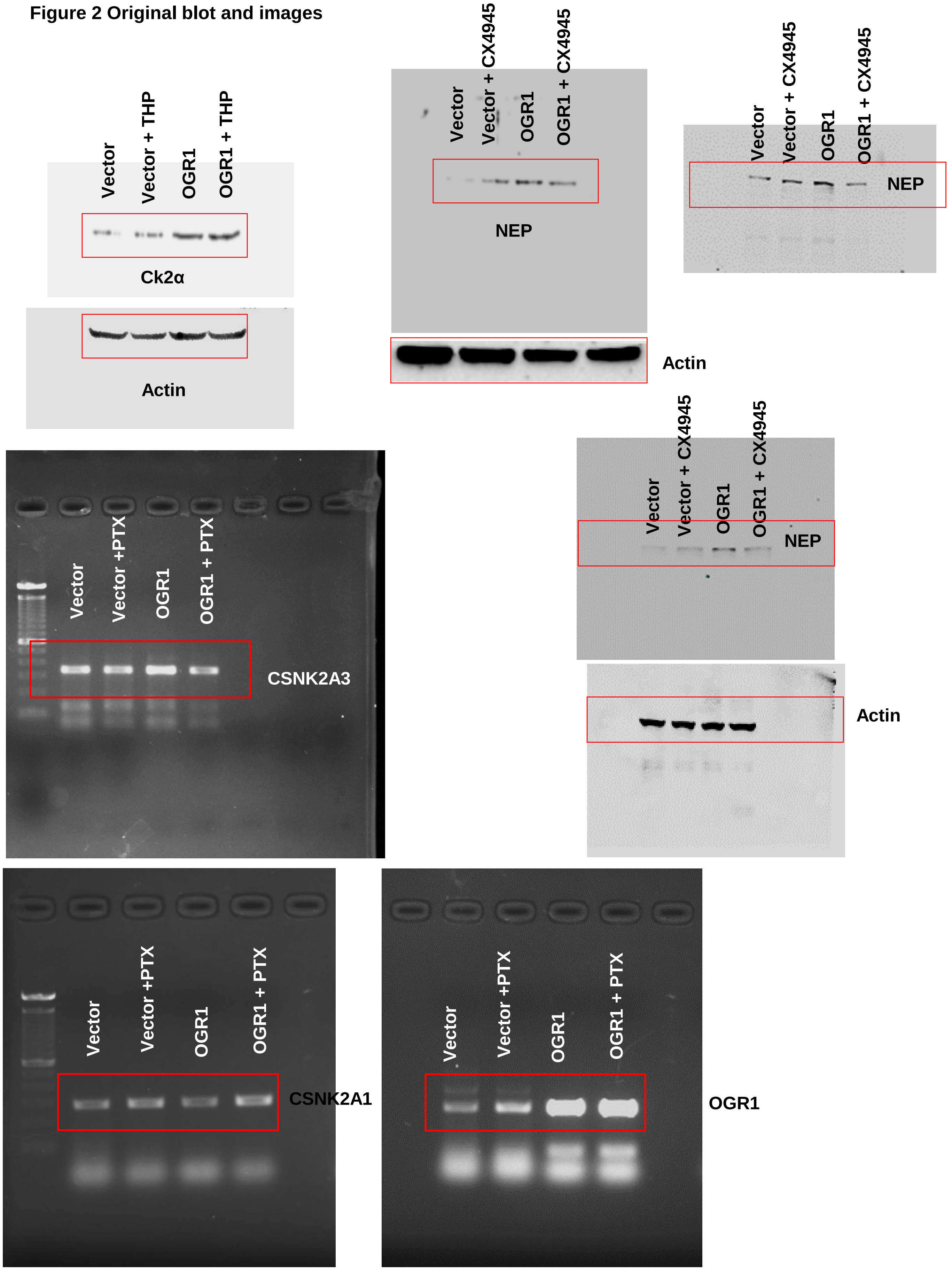

Ovarian cancer G protein-coupled receptor 1 inhibits A549 cells Migration through Casein kinase 2α intronless gene and Neutral endopeptidase

Adhikarimayum Lakhikumar Sharma [1, 2], Puyam Milan Meitei [1], Takhellambam Chanu Machathoibi [1], Naorem Tarundas Singh [1], Thiyam Ramsing Singh [1], Lisam Shanjukumar Singh [1*]

Figure 4A Original blot and images

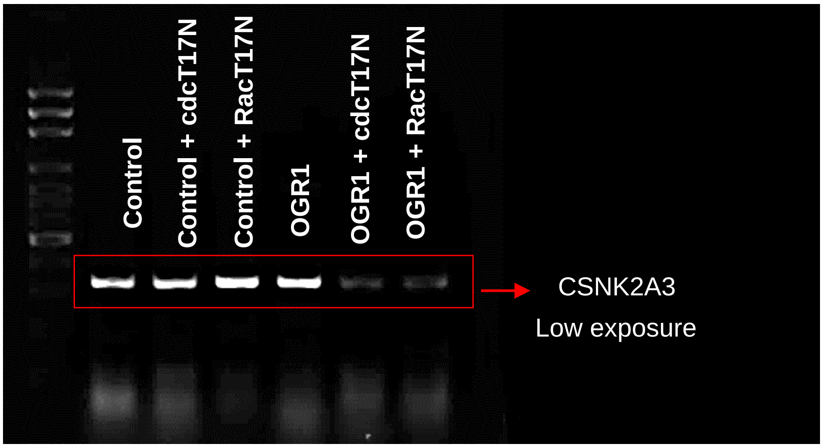

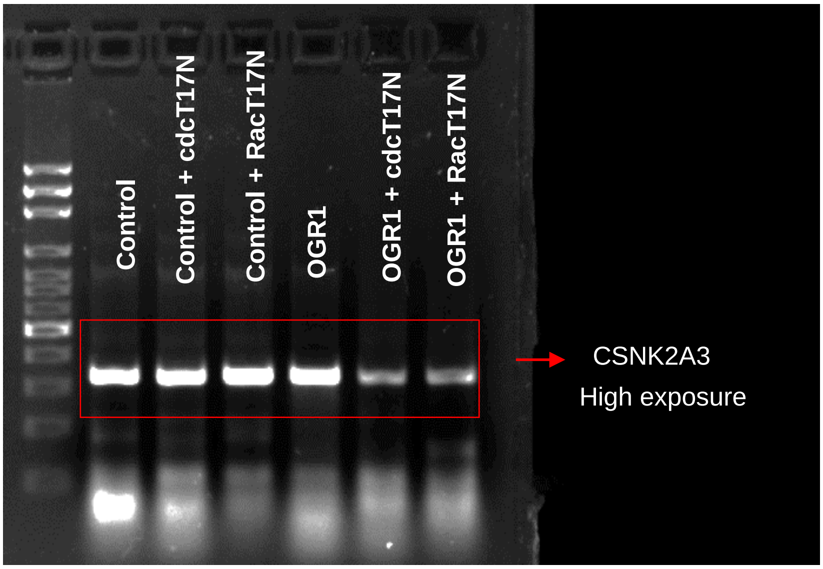

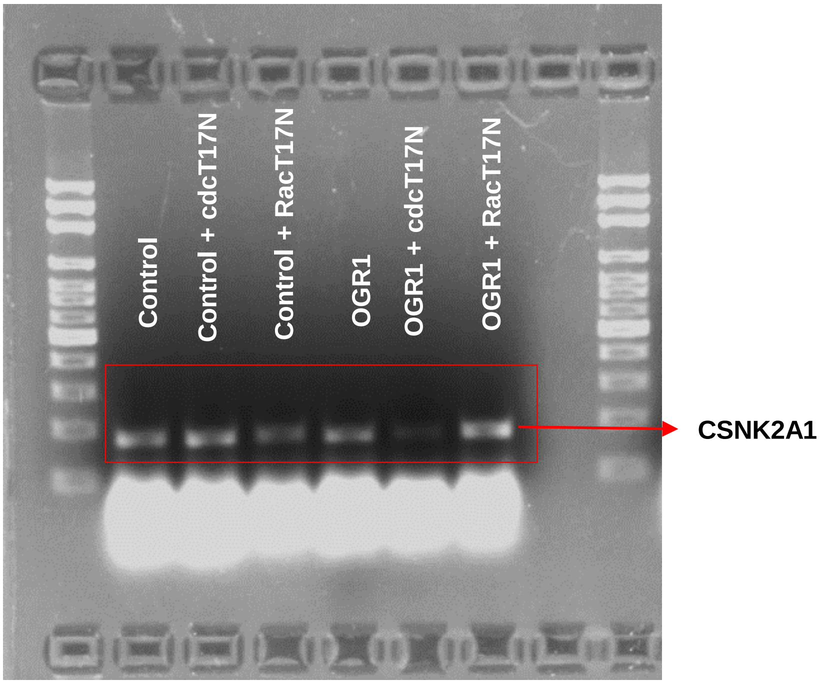

Ovarian cancer G protein-coupled receptor 1 inhibits A549 cells Migration through Casein kinase 2α intronless gene and Neutral endopeptidase

Adhikarimayum Lakhikumar Sharma [1, 2], Puyam Milan Meitei [1], Takhellambam Chanu Machathoibi [1], Naorem Tarundas Singh [1], Thiyam Ramsing Singh [1], Lisam Shanjukumar Singh [1 *]

Figure 4A continue RTPCR images

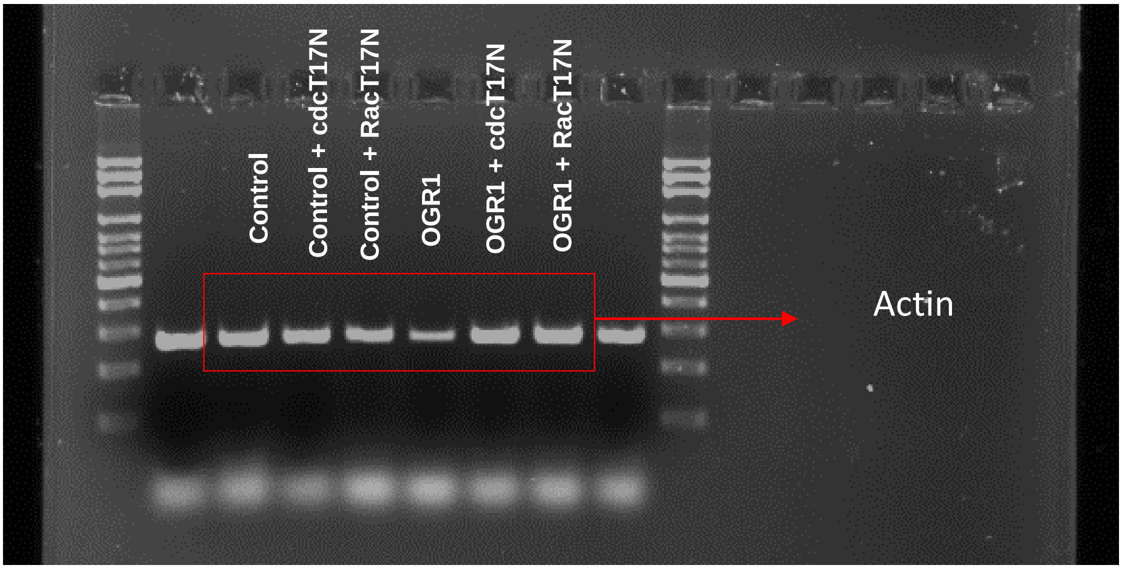

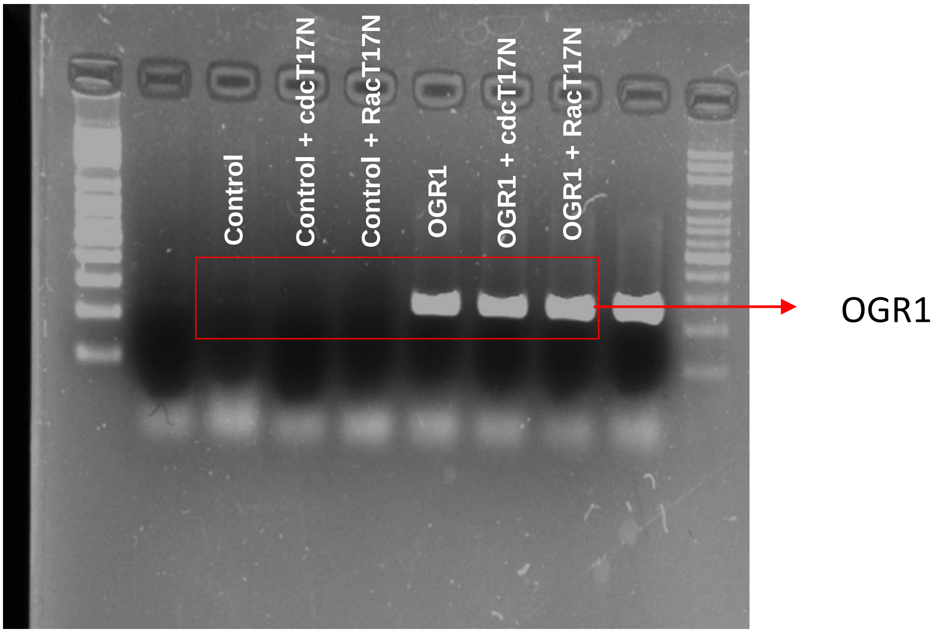

Ovarian cancer G protein-coupled receptor 1 inhibits A549 cells Migration through Casein kinase 2α intronless gene and Neutral endopeptidase

Adhikarimayum Lakhikumar Sharma [1, 2], Puyam Milan Meitei [1], Takhellambam Chanu Machathoibi [1], Naorem Tarundas Singh [1], Thiyam Ramsing Singh [1], Lisam Shanjukumar Singh [1 *]

Figure 4B Original blot and images

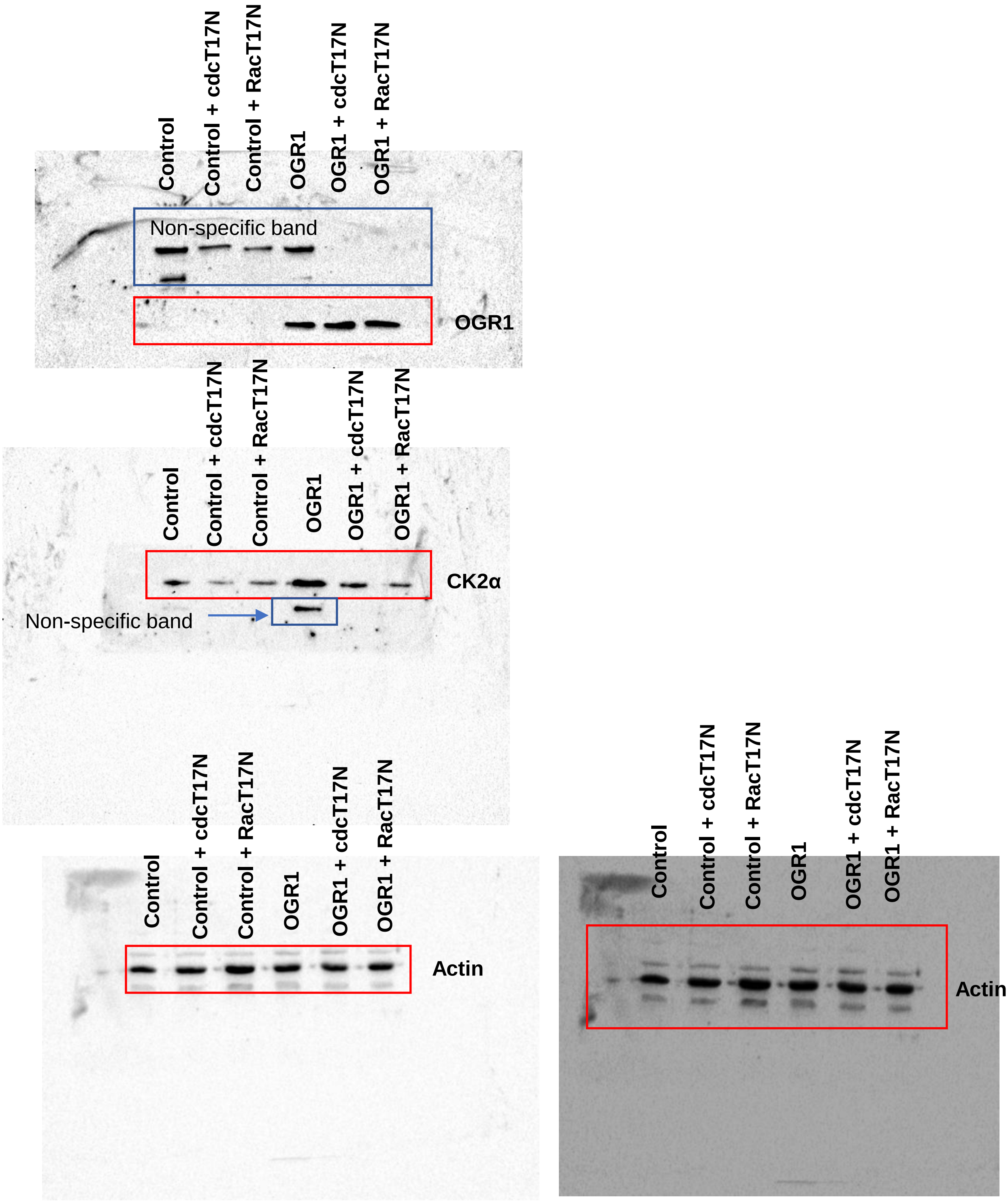

Immunoblot of OGR1, CK2α, and beta-actin after A549 cells were transfected with pcDNA3.1 vector and pcDNA3.1+OGR1 or co-transfected with dominant-negative mutants of cdc24 (cdcT17N) and Rac (RacT17N)

Ovarian cancer G protein-coupled receptor 1 inhibits A549 cells Migration through Casein kinase 2α intronless gene and Neutral endopeptidase

Adhikarimayum Lakhikumar Sharma [1, 2], Puyam Milan Meitei [1], Takhellambam Chanu Machathoibi [1], Naorem Tarundas Singh [1], Thiyam Ramsing Singh [1], Lisam Shanjukumar Singh [1 *]

Figure 4C Original blot and images

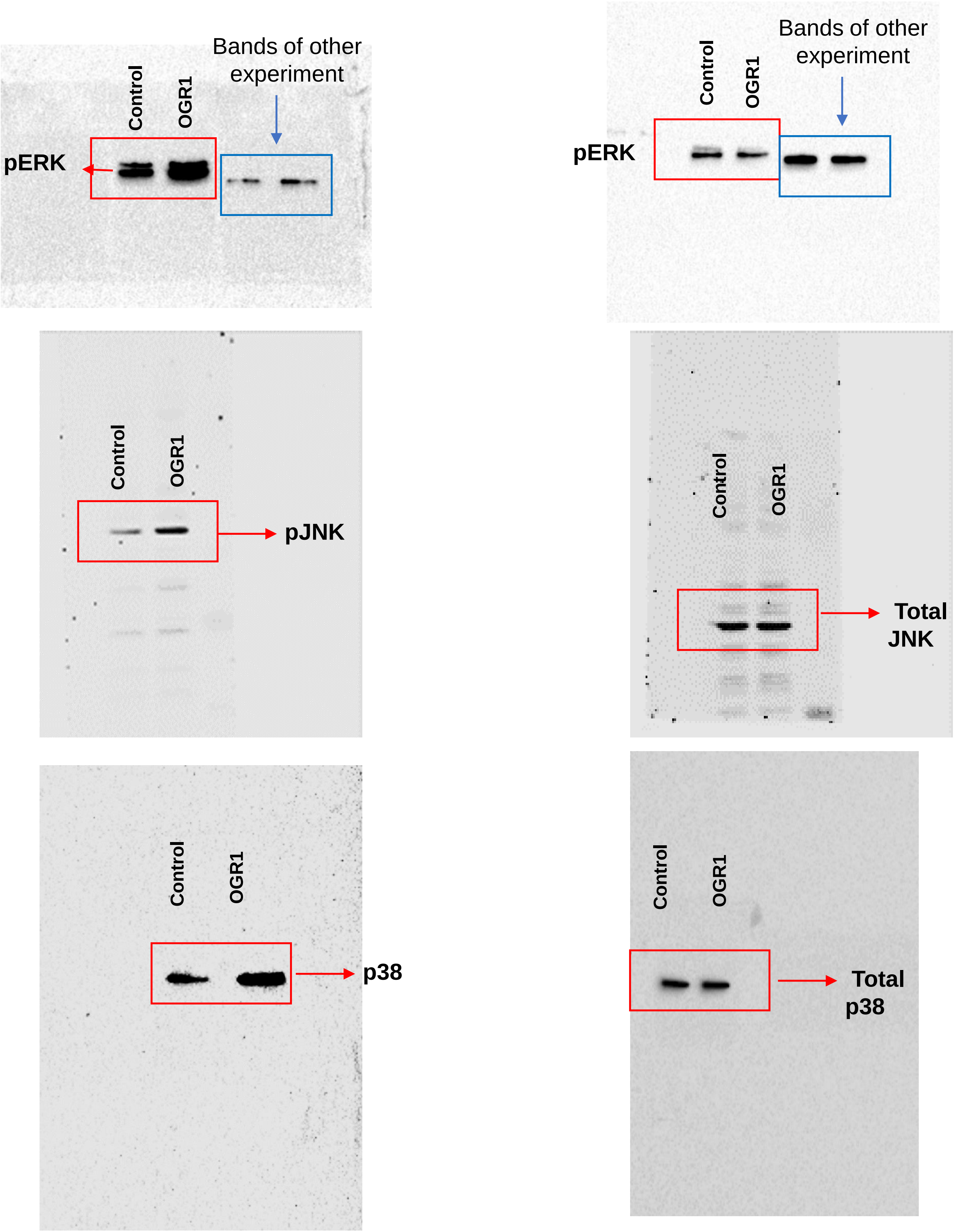

Ovarian cancer G protein-coupled receptor 1 inhibits A549 cells Migration through Casein kinase 2α intronless gene and Neutral endopeptidase

Adhikarimayum Lakhikumar Sharma [1, 2], Puyam Milan Meitei [1], Takhellambam Chanu Machathoibi [1], Naorem Tarundas Singh [1], Thiyam Ramsing Singh [1], Lisam Shanjukumar Singh [1 *]

Figure 4D Original RT PCR images

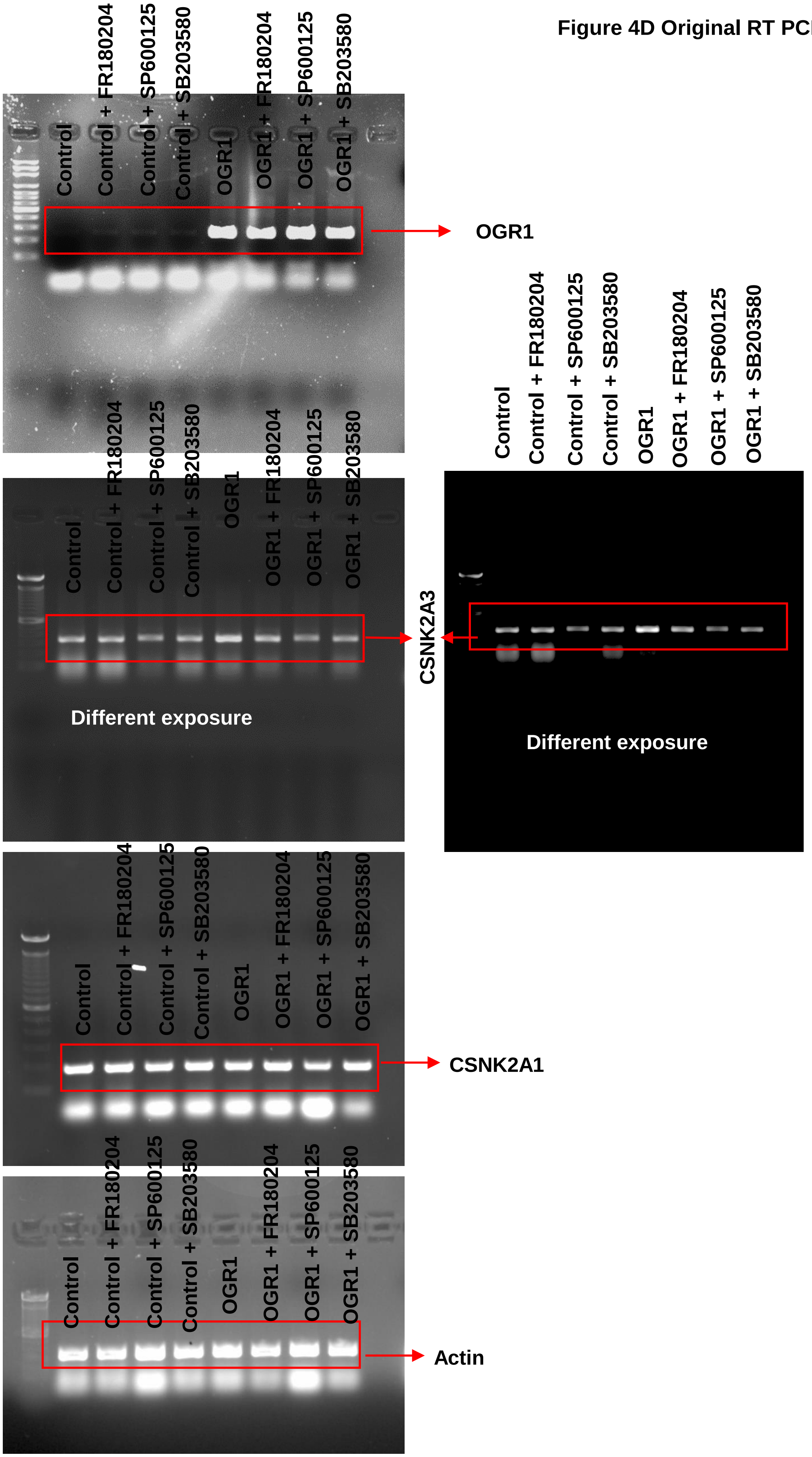

Ovarian cancer G protein-coupled receptor 1 inhibits A549 cells Migration through Casein kinase 2α intronless gene and Neutral endopeptidase

Adhikarimayum Lakhikumar Sharma [1, 2], Puyam Milan Meitei [1], Takhellambam Chanu Machathoibi [1], Naorem Tarundas Singh [1], Thiyam Ramsing Singh [1], Lisam Shanjukumar Singh [1 *]

Figure 4E Original blot and images

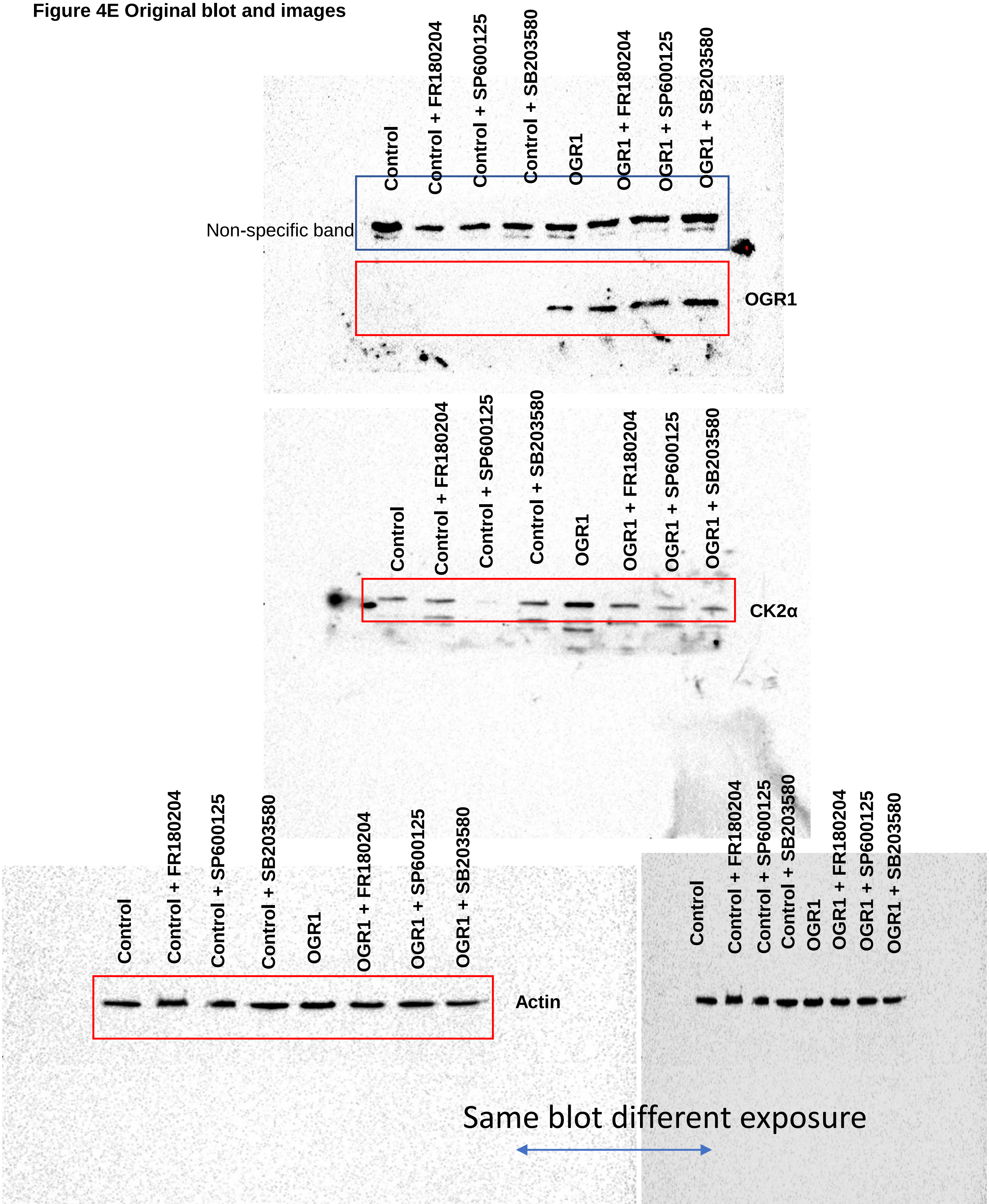

Immunoblot were performed to analyze CK2αP expression, respectively, in the presence of specific MAPK inhibitors. β-actin was used as a control for equal loading.

Ovarian cancer G protein-coupled receptor 1 inhibits A549 cells Migration through Casein kinase 2α intronless gene and Neutral endopeptidase

Adhikarimayum Lakhikumar Sharma [1, 2], Puyam Milan Meitei [1], Takhellambam Chanu Machathoibi [1], Naorem Tarundas Singh [1], Thiyam Ramsing Singh [1], Lisam Shanjukumar Singh [1 *]

Figure Supplementary file 1 Original blot and images

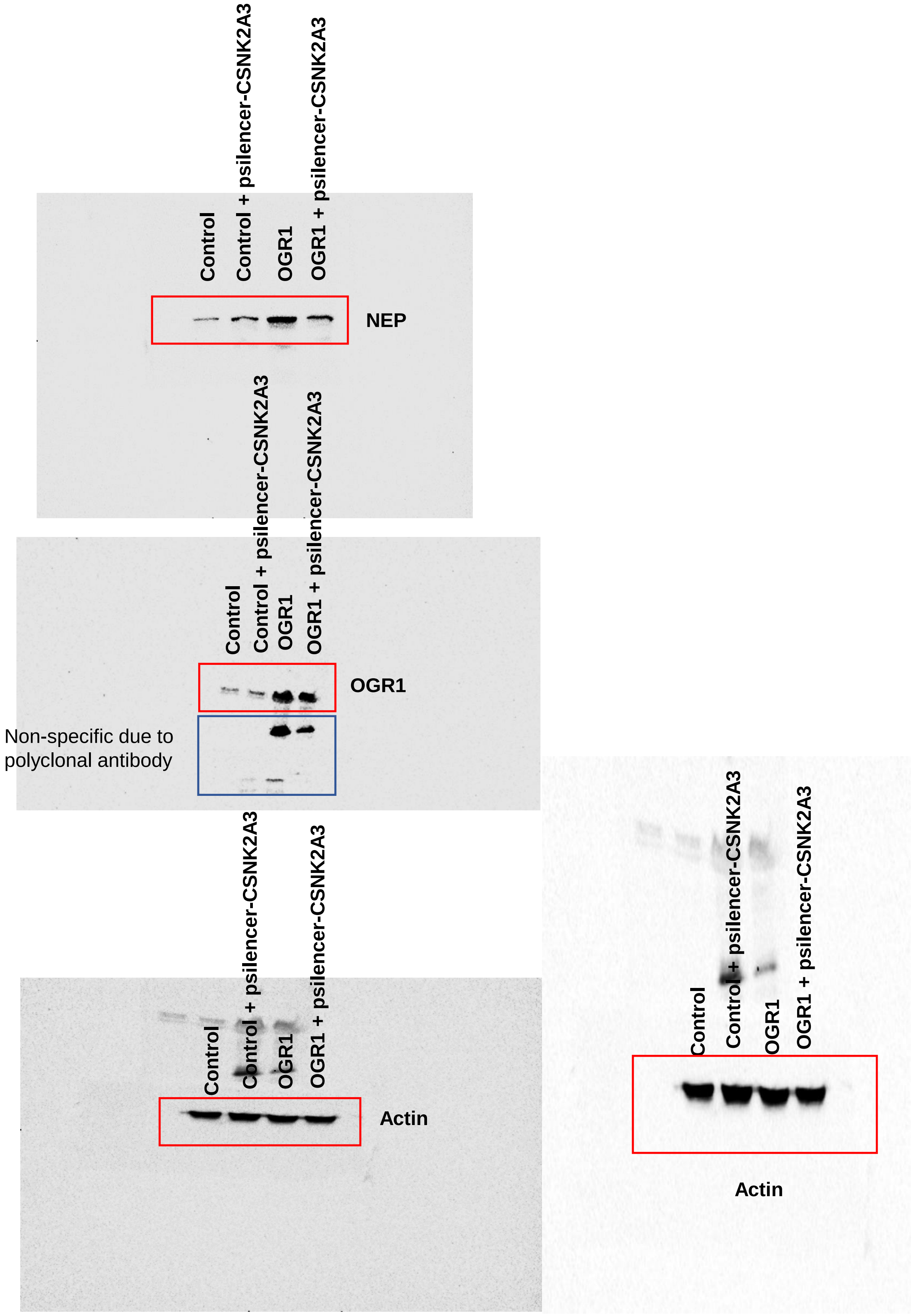

Supplement: Supplementary file 2 — Additional file 2. All the original gel images and western blot figures. [file 12885_2022_9257_MOESM2_ESM.pdf]
